# Ultrasonography Echotexture as a surrogate for Sialadenitis secondary to 131I Radioiodine Therapy for differentiated Thyroid Cancer: a review and metaanalysis

**DOI:** 10.6061/clinics/2020/e1843

**Published:** 2020-10-05

**Authors:** Graziele Aparecida Simões Lima, Rossana Verónica Mendoza López, Gislaine Aparecida Ozório, Ricardo Miguel Costa de Freitas, Jose Willegaignon, Marcelo Tatit Sapienza, Maria Christina Chammas, George Barberio Coura-Filho

**Affiliations:** I Instituto do Cancer do Estado de Sao Paulo (ICESP), Hospital das Clinicas HCFMUSP, Faculdade de Medicina, Universidade de São Paulo, São Paulo, SP, BR; II Centro de Investigacao Translacional em Oncologia, Instituto do Cancer do Estado de Sao Paulo (ICESP), Hospital das Clinicas HCFMUSP, Faculdade de Medicina, Universidade de Sao Paulo, Sao Paulo, SP, BR; III Serviço de Radiologia, Instituto do Cancer do Estado de Sao Paulo, Hospital das Clinicas HCFMUSP, Faculdade de Medicina, Universidade de Sao Paulo, Sao Paulo, SP, BR; IV Serviço de Medicina Nuclear, Instituto do Cancer do Estado de Sao Paulo, Hospital das Clinicas HCFMUSP, Faculdade de Medicina, Universidade de Sao Paulo, Sao Paulo, SP, BR; V Centro de Medicina Nuclear, Instituto de Radiologia (InRad), Hospital das Clinicas HCFMUSP, Faculdade de Medicina, Universidade de Sao Paulo, Sao Paulo, SP, BR; VI Instituto de Radiologia (InRad), Hospital das Clinicas HCFMSUP, Faculdade de Medicina, Universidade de Sao Paulo, Sao Paulo, SP, BR

**Keywords:** Differentiated Thyroid Cancer, Radioiodine, Major Salivary Glands, Ultrasonography, Sialadenitis

## Abstract

To systematically review and analyze the medical literature to assess ultrasonography echotexture changes in thyroid cancer patients for the detection of chronic sialadenitis caused by radioiodine therapy. Methods: Sources were retrieved from PubMed, Scopus, EMBASE and LILACS through November 2018. All studies that assessed ultrasonographic features before ^131^I administration and at 12 months after ^131^I administration were selected. After data extraction, statistical analysis was performed by using Stata software. Results: From a total of 435 studies, 4 studies involving 665 patients were considered eligible, and echotexture heterogeneity was found with a significant difference. Conclusions: Ultrasound echotexture may detect chronic sialadenitis secondary to salivary radioiodine therapy.

## INTRODUCTION

Differentiated thyroid carcinoma (DTC) is the most common form of thyroid cancer (TC) and is responsible for more than 80% of all TC cases. Standard treatment for DTC usually includes surgical resection followed by radioiodine therapy with ^131^I (RIT) in selected cases for ablative or adjuvant purposes (1-4).

Salivary glands are also capable of concentrating radioiodine, and their function can be affected by radiation, resulting in sialadenitis, defined as inflammation of the salivary glands (5,6). The initial signs and symptoms of sialadenitis vary but may include swelling and pain in the salivary glands with possible subsequent dry mouth. When it presents as chronic inflammation, sialadenitis may cause gland atrophy and the replacement of normal tissue by fibrous tissues (7,8).

Usually, salivary glands have a homogeneous echotexture on ultrasound (US), but in chronic sialadenitis, the echotexture is generally heterogeneous, and the parotid glands are more severely affected by radiation compared to other salivary glands (8,9).

Although US is routinely performed to evaluate DTC recurrence in patients who undergo surgical resection followed by RIT, it may also be used as an auxiliary tool for sialadenitis diagnosis, aiding clinical decision making during DTC follow-up (5).

The aim of this study was to systematically review and analyze the medical literature to assess US echotexture changes in DTC patients for the detection of chronic sialadenitis caused by RIT.

## MATERIALS AND METHODS

### Eligibility Criteria

Studies were included for analysis if they met the following criteria: patients who (1) had undergone total or near-total thyroidectomy as an initial treatment, (2) were treated with ^131^I for the first time after surgery, (3) had clearly defined ^131^I administered activity, and (4) underwent neck US follow-up before ^131^I administration and at least 12 months after ^131^I administration with identification of the major salivary glands and characterization of their homogeneous or heterogeneous echotexture.

Studies were excluded for any of the following reasons: if the study was not published as a full article (i.e., conference presentations), if the study contained pediatric data, duplicate publications of data from the same patients, if the study did not evaluate the echotexture of the major salivary glands through ultrasonography features, and if in the study the patients had not undergone ultrasound exams evaluating the salivary gland areas before and 12 months post radioiodine therapy.

The diagnosis of chronic sialadenitis was defined as heterogeneity in the echotexture of the salivary glands on ultrasonography at least 12 months after RIT.

### Data Sources

A systematic literature review was conducted. Studies were retrieved from the following electronic databases: PubMed (1982 to November 2018), Scopus (1974 to November 2018), EMBASE (1989 to November 2018) and LILACS (2015 to November 2018).

### Search Strategy and Data Extraction

Two investigators independently searched the electronic databases. The search strategy for PubMed was conducted by combining the terms “Iodine Radioisotopes” AND (“Salivary Glands” OR Sialadenitis) AND (“Thyroid Neoplasms” OR “Thyroid Carcinoma”), Filters: Humans, Adult: 19+ years. For Scopus, the terms “Iodine Radioisotopes” AND (“Salivary Glands” OR sialadenitis) AND (“Thyroid Neoplasms” OR “Thyroid Carcinoma”) AND (adult* OR aged OR “Aged, 80 and over”) AND human*. Searches using EMBASE and LILACS were conducted by combining the terms “Iodine Radioisotopes” AND (“Salivary Glands” OR sialadenitis) AND (“Thyroid Neoplasms” OR “Thyroid Carcinoma”).

The searches were limited to articles published in English. The investigators inspected the titles and abstracts of the citations to identify relevant publications and obtained their full text for screening against the inclusion/exclusion criteria.

A standardized data collection form was used. From each eligible trial, the following data were compiled in the database: author names, year of publication, country of origin, number of participants, sex and age distribution, follow-up time, definition of salivary glands evaluated, and administered radioiodine activity.

### Quality Assessment

The quality of the studies was assessed according to a checklist based on the proposal by the preferred reporting items for systematic reviews and meta-analyses (PRISMA) (10).

### Statistical Analysis

The statistical analysis was performed, and a forest plot was generated using Stata software (Version 11 for Windows; StataCorp LLC, College Station, Texas, United States of America). The heterogeneity I^2^ test and numerical summary measurements were calculated. Both heterogeneity and confidence intervals were assessed as interesting measures in the meta-analysis results. The level of significance was 5% for all hypothesis tests.

## RESULTS

**Figure 1:**
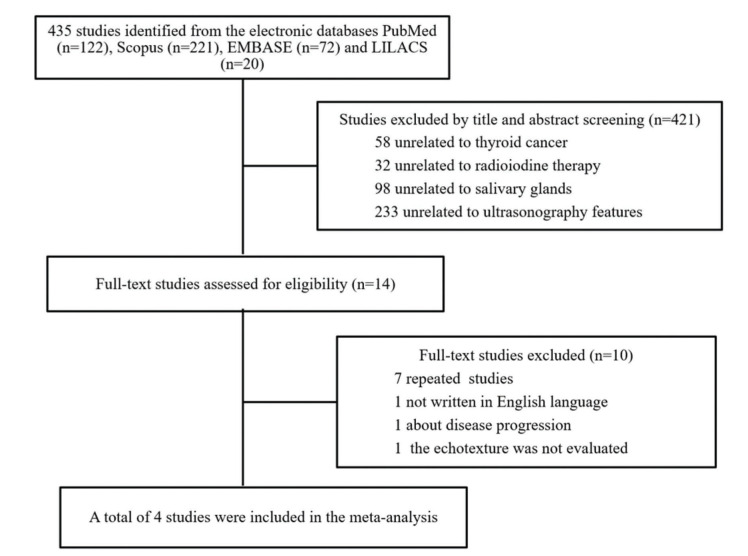
Flowchart diagram of the study selection.

A total of 435 abstracts were found through the literature search. According to the eligibility criteria, 421 were excluded, and fourteen were identified as eligible for full-text review. However, ten full texts were excluded for different reasons: seven studies were duplicated, one was not written in English, one study evaluated progressive disease, and one did not evaluate the echotexture of the salivary glands. All of the four remaining studies provided adequate data for the analysis. The selection of the articles is shown in the flowchart in Figure 1.

All included studies were published recently (2013-2016). A total of 655 patients were included, and the sample size per study ranged from 43 to 256 patients with a female sex predominance.

Although different radioiodine-administered activities were used in these studies (from 1100 to 44400 MBq) and the time of follow-up was different, all of them evaluated the ultrasonography echotexture of the salivary glands before and after radioiodine therapy, as shown in Table 1.

All four studies provided adequate data for the analysis. The ablation radioiodine dose and changes in echotexture of the parotid salivary gland data from each included study are displayed in Table 2. Two studies showed a significant difference, and two studies did not show a significant difference.

**Figure 2:**
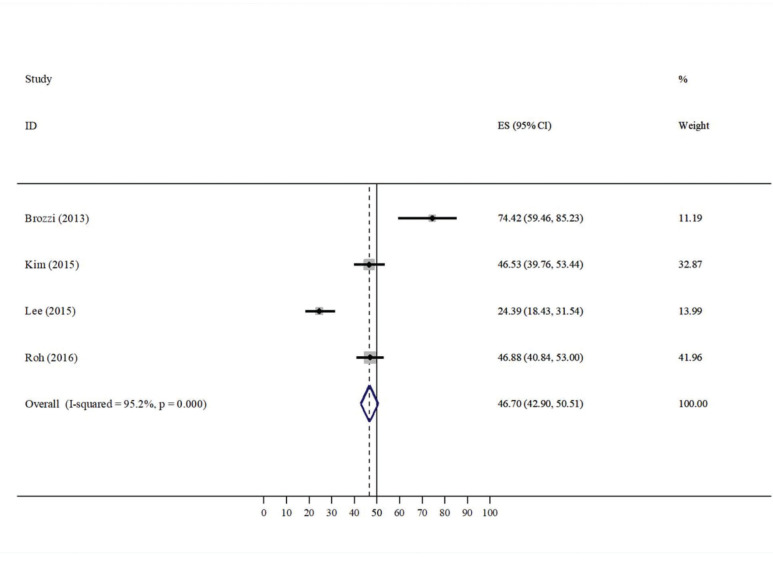
Comparison of the ultrasonographic features after remnant ablation.

Heterogeneity was found in the pooled analysis, and the difference was significant (I^2^ =94.7%, *p*<0.001; Figure 2), so a random-effects model was used for further data evaluation. Comparing all of the included studies, 46.70% of patients presented with changes in their salivary glands after RIT.

## DISCUSSION

Ablation or adjuvant therapy of the residual thyroid or residual DTC tumors by oral administration of ^131^I after total thyroidectomy surgery is well established in the management of patients with DTC. The American Thyroid Association (ATA) currently considers RIT based on disease recurrence rates and recommends it for all high-risk patients and selected intermediate-risk patients but not for low-risk patients. The RIT recommendations are believed to be associated with improved overall survival by reducing the risk of DTC recurrence and mortality (4,11-13).

Recently, many studies have reported side effects on salivary glands as the most frequent complication post-RIT, mainly of the major salivary glands. According to the medical literature, this side effect occurs due to the high radiosensitivity and high turnover rate of epithelial cells in salivary glands and can result in sialadenitis. To monitor the side effects post radioiodine treatment, US has been used as a tool to infer sialadenitis damage post-RIT due to its capacity to identify a heterogeneous echotexture of the major salivary glands (2,14-16).

There is no register of previous systematic reviews that evaluated chronic sialadenitis secondary to RIT by ultrasonography echotexture of the salivary glands. During the past six years, only four studies were published investigating the potential role of US. In these reports, three studies evaluated South Korean patients, and one study evaluated Italian patients. In all of the studies, there was a predominance of women. The minimum ablation radioiodine dose was the same in all of the studies, but all were performed considering different ranges of radioiodine-administered activity. Although these four studies presented with different sample sizes, as demonstrated before, the adverse effects of the treatment were evaluated by ultrasonography features, and heterogeneous echotexture of the salivary glands was found.

This systematic review was performed to better understand the impact of RIT on salivary glands by including studies that evaluated the echotexture of the salivary glands post-RIT by US. As shown in the results section, significant echotexture heterogeneity was found in the pooled analysis, consistent with the literature. In 2015, Lee et al. evaluated the symptoms of the side effects of RIT by analyzing the imaging findings of salivary glands in 164 patients and demonstrating echotexture heterogeneity in 24.39% of patients, with a statistical association (*p*<0.001) (5). Brozzi et al. performed the same evaluation in 2013 and showed echotexture heterogeneity of major salivary glands in 74.42% of patients, with a statistical association (*p*=0.002). However, the sample size of their study was only 43 patients, limiting the data interpretation (13). Kim performed a study in 202 patients from January 2009 to December 2009 and found echotexture heterogeneity in major salivary glands in 46.53%, with no statistical association (*p*=0.325) (14). Roh et al. evaluated 256 patients from January 2011 to December 2011 and reported heterogeneity in major salivary glands in 46.8% of patients, with no statistical association (*p*=0.325) (15).

The common post-RIT ultrasonography features in all of these studies also included a coarse echotexture and a decreased size of the salivary glands. Lee et al. have found internal ductal dilatation in 3 (7.5%) of the 40 patients who had changes in salivary glands posttherapy, but this feature was not evaluated by the other authors, although it can be useful in identifying chronic sialadenitis (5).

Overall variations in the statistical associations among the studies could be caused mainly by different sample sizes and different ranges of administered ^131^I activity. The present meta-analysis allows for a better understanding of the reasons behind the inconsistent results previously reported in the medical literature and enhances comprehension of the role of US in the evaluation of chronic sialadenitis post-RIT.

Alexander et al. performed a standard study on the effects of RIT. A total of 203 patients were included in this study, and a diagnosis of sialadenitis was made for 33% of patients, with a statistical association (*p*=0.004), but the sialadenitis diagnosis was not made through auxiliary US features (16).

As previously described, damage to the salivary glands secondary to RIT is activity dependent, agreeing with the results found in all the studies included in our meta-analysis. Kim evaluated the correlation between the prevalence of post-RIT salivary gland damage and radioiodine activity and found a significant association (*p*=0.0001) (14). Lee et al. also found a significant difference in the development of sialadenitis between patients who received high radioiodine activity, such as 3700 MBq and 5550 MBq (*p*<0.001) (5). In agreement, Roh et al. demonstrated changes in US features of the parotid gland were correlated more with higher RIT activities than with lower activities (*p*<0.0001) (15). Brozzi et al. did not assess damage to the salivary glands post-RIT according to the radioiodine activity (13).

As reported in previous studies, side effects such as xerostomia, swelling, and dry mouth are common post-RIT as well. Lee et al. found swelling (60.5%), xerostomia (53.9%) and pain (23.7%) in 76 of the 164 patients who complained of symptoms related to salivary glands dysfunction posttherapy (5). According to Brozzi et al., 32 patients who underwent RIT did not complain of any symptoms related to RIT, but among the 43 treated patients, there was swelling in 4 (9%), dry mouth (21%) and xerostomia (6.9%) (13). Roh et al. showed xerostomia in 85 (33%) of the 256 evaluated patients, and Kim did not evaluate any symptoms related to RIT, which was a limitation of that study (14,15).

In conclusion, the present meta-analysis summarizes all available evidence about the side effects caused by RIT on salivary glands in patients with DTC through echotexture evaluation. US might serve as a potential tool in post-RIT follow-up to characterize chronic sialadenitis.

Currently, US exams have been used in many studies as an auxiliary tool in sialadenitis diagnosis through observing changes in the echotexture of the salivary glands, and the present study complemented previous reviews. Nevertheless, the limitations of this meta-analysis include the small sample of the evaluated population, the retrospective design of the studies and the predominance of Asian populations. Thus, further prospective studies correlating the US findings with clinical symptoms and examining other populations may be necessary to better understand the full role of US in post-RIT sialadenitis evaluation.

## AUTHOR CONTRIBUTIONS

Lima GAS, Ozório GA and Coura-Filho GB participated in the study design, data collection and analysis, and manuscript writing. López RVM participated in the study design, statistical analysis and manuscript writing. Freitas RMC, Willegaignon J, Sapienza MT and Chammas MC participated in the data analysis and manuscript review.

## Figures and Tables

**Table 1:** Characteristics of the eligible studies.

Study (year)	Country	Study design	Sample	Sex F/M	RIT activity, (MBq)	Ultrasonographic features evaluated	Follow-up time (mo)
Brozzi (2013)	Italy	RS	43	28/15	1110 to 44400	HO *vs* HE	6-144
Kim (2015)	South Korea	RS	202	172/30	1110 to 6660	HO *vs* HE	3-70
Lee (2015)	South Korea	RS	164	130/34	1110 to 7400	HO *vs* HE	6-27
Roh (2016)	South Korea	RS	256	223/33	1110 to 6660	HO *vs* HE	12-50

RS= Retrospective Study, F= Female, M= Male, HO = Homogeneous, HE = Heterogeneous; mo = Months.

**Table 2:** Related data from the included studies.

		Heterogeneous (%)	
Study (year)	RIT activity (MBq)	(95% CI)	*p*
Brozzi (2013)	1110 *vs.* 44400	74.42 (59.46-85.23)	0.002
Kim (2015)	1110 *vs.* 3700 *vs.* 5550 *vs.* 5920 *vs.* 6660	46.53 (39.76-53.44)	0.325
Lee (2015)	1110 *vs.* 3700 *vs.* 5550 *vs.* 8140	24.39 (18.43-31.54)	< 0.001
Roh (2016)	1100 *vs.* 2220 *vs.* 3700 *vs.* 4810 *vs.* 5550 *vs.* 5920 *vs.* 6660	46.88 (40.84-53.00)	0.318
